# Dynamic Prediction of Survival Outcomes in Multiple Myeloma

**DOI:** 10.3390/cancers18172864

**Published:** 2026-09-04

**Authors:** Kelly Quek, Cindy H. Lee, Yang Zhang, Barbara J. McClure, Runzhe Chen, Hamish S. Scott, Kate Vandyke, Andrew C. W. Zannettino, Chung Hoow Kok

**Affiliations:** 1 Accenture, Singapore 179101, Singapore; 2 Department of Haematology, Royal Adelaide Hospital, Central Adelaide Local Health Network, Adelaide, SA 5000, Australia; cindy.lee3@sa.gov.au; 3 School of Medicine, College of Health, Adelaide University, Adelaide, SA 5000, Australia; yang.zhang03@adelaide.edu.au; 4 Department of Genetics and Molecular Pathology, SA Pathology, Adelaide, SA 5000, Australia; hamish.scott@sa.gov.au; 5 School of Pharmacy and Biomedical Science, College of Health, Adelaide University, Adelaide, SA 5000, Australia; kate.vandyke@adelaide.edu.au (K.V.); andrew.zannettino@adelaide.edu.au (A.C.W.Z.); 6 Centre for Cancer Biology, College of Health, Adelaide University, Adelaide, SA 5000, Australia; barbara.mcclure@adelaide.edu.au; 7 Department of Health Promotion and Behavioral Sciences, School of Public Health, The University of Texas Health Science Center at Houston, Houston, TX 77030, USA; runzhe.chen@outlook.com; 8 Precision Cancer Medicine Theme, South Australian Health & Medical Research Institute (SAHMRI), Adelaide, SA 5000, Australia

**Keywords:** multiple myeloma, myeloma-specific survival, prognostication, transcriptomics, gene expression, molecular pathways, Bayesian network, precision oncology, dynamic risk stratification

## Abstract

**Background**: Multiple myeloma (MM) remains an incurable plasma cell malignancy characterized by marked clinical heterogeneity. Existing prognostic frameworks, including the International Staging System (ISS) and FISH-defined cytogenetic risk, are anchored at diagnosis and do not capture the evolutionary dynamics of disease or treatment response, leaving an unmet need for risk models that retain prognostic validity longitudinally. **Methods**: Using transcriptomic data from 762 CD138-selected MM plasma cells from newly diagnosed patient samples in the MMRF CoMMpass study (NCT01454297), we computed single-sample pathway activity scores for 469 curated cancer-relevant pathways (MSigDB Hallmark; Reactome) and learned a Bayesian causal network linking pathway activity to survival. The model was validated in five independent diagnostic cohorts (*n* = 1255) and in two independent treatment and relapsed/refractory cohorts (*n* = 319). Longitudinal risk tracking was additionally assessed in a 46-patient subset of the discovery cohort with serial pre- and post-treatment sampling. **Results**: The network identified five pathways associated with survival: unfolded protein response (UPR), FLT3 signaling through SRC family kinases, G2M DNA replication checkpoint, metabolism of selenium compound (SeMet), and nicotinate metabolism. The composite survival score stratified patients into high-risk (*n* = 76; 10%) and standard-risk groups with markedly divergent survival (median 1170 days vs. not reached; *p* < 0.0001). The score remained an independent prognostic factor after adjustment for age, sex, ISS stage, and *KRAS*, *TP53*, and *UBR5* mutational status (HR 4.93; 95% CI 2.96–8.19; *p* < 0.001), and replicated across all five external diagnostic cohorts. Critically, the model retained prognostic discrimination in previously treated (GSE57317; *p* < 0.0001) and relapsed/refractory (GSE9782; *p* < 0.0001) settings, and patients transitioning from standard- to high-risk between serial samples exhibited significantly inferior survival compared to standard-risk patients. **Conclusions**: This pathway-based Bayesian network provides a reproducible, dynamically applicable risk model for MM that captures information complementary to ISS and FISH-defined cytogenetics. The framework supports longitudinal patient monitoring and may inform trial enrichment strategies and closer surveillance for high-risk subpopulations.

## Simple Summary

Multiple myeloma is a cancer of plasma cells that can behave very differently between patients. Current risk assessment is mainly based on tests performed at diagnosis, but the disease can change over time and in response to treatment. We therefore developed a gene expression-based score derived from cancer-relevant biological pathways to predict survival risk both at diagnosis and later in the disease course. The risk score was evaluated across more than 2000 patient samples from several independent cohorts. It consistently identified patients with poorer survival, improved prediction of survival risk beyond established clinical staging, and remained informative in previously treated and relapsed or treatment-resistant disease. Patients with changes in risk status from standard to high risk after treatment had poorer outcomes. If confirmed prospectively, this approach could support repeated prediction of survival risk and closer monitoring of patients at higher risk.

## 1. Introduction

Multiple myeloma (MM) is the second most common hematological malignancy and remains incurable in the majority of patients despite substantive therapeutic advances over the past two decades [1,2]. Five-year relative survival has risen to approximately 58%, but outcomes vary widely: a substantial subset of patients experience early relapse and rapid progression to refractory disease, while others achieve durable responses [2]. The molecular determinants of this heterogeneity are incompletely defined, and clinically deployed risk frameworks remain limited in scope and temporal applicability.

The current clinical risk stratification in MM relies predominantly on the Revised International Staging System (R-ISS) [3], which integrates serum β2-microglobulin, albumin, lactate dehydrogenase, and FISH-detected high-risk cytogenetic abnormalities (del(17p), t(4;14), t(14;16)). While prognostically useful at diagnosis, these classifiers are static; they do not accommodate the clonal evolution, treatment-induced selection pressures, and emergent resistance mechanisms that characterize MM progression [4]. Gene expression-based classifiers such as the UAMS-70 [5] and EMC-92 [6] signatures have improved upon clinical staging but are similarly anchored to baseline disease biology and have shown variable performance across cohorts and treatment eras. Overexpression of single genes, for example, *DSG2* [7,8] and *PHF19* [9], can also be predictive of poor prognosis but is not currently incorporated in clinical staging.

Within a precision oncology framework, two limitations are particularly consequential. First, single-gene or signature-level classifiers often reduce complex transcriptional programs into a single score, potentially obscuring the modular pathway-level biology that underlies disease behavior. Second, very few existing tools have been designed or formally validated for serial application across the patient journey. This represents a gap in translational practice given that risk stratification is increasingly informed by molecular re-evaluation [10].

We hypothesized that a Bayesian network constructed using patient-level pathway activity scores would (i) identify a parsimonious set of biologically interpretable pathways with direct associations with survival, (ii) yield a composite score that stratifies risk independently of ISS and recurrent driver mutations, and (iii) retain prognostic validity when applied dynamically to post-treatment and relapsed disease states. Bayesian networks are well-suited to this task because they explicitly model conditional dependencies between variables and provide an interpretable framework for inference. This distinguishes them from many black-box machine-learning approaches, which may be less transparent and therefore less amenable to translation to clinical practice [11,12].

We propose this score for two related but distinct uses: as a baseline prognostic tool at diagnosis, complementary to ISS and cytogenetic risk, and as a serial monitoring tool to identify changes in molecular risk during the disease course. We do not propose the score for treatment selection, as no data presented here evaluate a score-by-treatment interaction. Its potential clinical applications include risk-based trial enrichment and identification of patients who may warrant closer surveillance, both of which require prospective validation.

## 2. Materials and Methods

### 2.1. Patient Cohorts and Data Sources

Discovery cohort transcriptomic, clinical, and somatic mutation data were obtained from the MMRF CoMMpass study (NCT01454297; https://research.themmrf.org; https://portal.gdc.cancer.gov and was accessed 2 June 2025), comprising 762 CD138-selected newly diagnosed bone marrow patient samples with paired RNA-sequencing (FPKM-normalized) and outcome data [13]. Myeloma-specific survival was defined as time from diagnosis to myeloma-attributable death or last follow-up; non-myeloma deaths were censored. Of 153 deaths, 84 (55%) were myeloma-related and used as events.

Five independent diagnostic validation cohorts (total *n* = 1255) and two independent post-treatment and relapsed/refractory cohorts (*n* = 319) were assembled from public repositories (Tables S1 and S2 detail accession, sample size, treatment, and platform). A further 46-patient subset of the discovery cohort with serial samples collected before and after treatment was analyzed separately for longitudinal risk tracking. The normalized gene expression datasets were obtained from the ArrayExpress database with accession number E-MTAB-4032 [14,15] and the NCBI GEO database with accession numbers GSE136337 [14], GSE19784 [16], GSE24080-TT2 [17,18,19], and GSE24080-TT3 [18,19,20] with overall survival information. Where duplicate gene-level probes were present, expression values were averaged prior to downstream analysis.

Serial molecular profiling samples within the MMRF CoMMpass study [21] were collected at diagnosis and, when available, at the time of suspected complete response, recurrence, or disease progression.

### 2.2. Mutational Analysis

MMRF somatic mutation data were obtained from the GDC portal. Non-synonymous coding variants annotated in the COSMIC Cancer Gene Census, with tumor and normal depth ≥ 10 reads, were retained. Genes mutated in fewer than 10 patients (~1.3%) were excluded. The only genes meeting statistical significance (*p* < 0.05) for differential mutation frequency between risk groups were subjected to multivariable Cox regression.

### 2.3. Pathway Curation and Activity Scoring

Molecular pathways were sourced from expert-curated repositories: MSigDB Hallmark and Reactome (*n* = 1701 pathways) [22,23]. We retained 635 pathways relevant to molecular signaling, immune response, and cell-cycle regulation under the cancer-relevant framework. Pathway gene sets exhibiting Pearson correlation r > 0.9 were collapsed to mitigate redundancy, yielding 469 non-redundant pathways. Per-patient pathway activity scores were computed using single-sample gene set enrichment analysis (ssGSEA) implemented in the *hacksig* R package [24], with combined Z-score normalization as described by Lee et al. [25]. Briefly, gene expression values were mean-centered and scaled by their standard deviation across samples (gene-wise Z-scores); for each sample and signature, Z-scores of constituent genes were summed and divided by the square root of the signature size. Gene-wise Z-scores were calculated separately within each dataset prior to pathway-level aggregation, standardizing pathway activity scores within each dataset; no additional batch-effect correction across platforms was applied because the cohorts were analyzed independently.

### 2.4. Bayesian Network Construction

A Bayesian network was constructed using the *bnlearn* R package [11] over 469 pathway activity variables and a survival outcome node. Network structure was learned via score-based hill-climbing search optimizing the Bayesian Information Criterion (BIC); the algorithm was initialized with an empty graph and iteratively added, removed, or reversed edges until no further improvement in BIC score was achieved. Conditional probability distributions for each node were estimated by maximum likelihood given the learned structure. Per-patient survival scores were generated using the *bn.predict* function. Network visualization was rendered using the *bnviewer* package. To assess the stability of the learned network structure given the number of candidate nodes relative to the number of outcome events, bootstrap resampling (R = 100) was performed using bnlearn::boot.strength(). The hill-climbing search using the BIC score (score = “bic-g”) was refitted in each resampled dataset, and the frequency with which each of the five reported directed edges to the survival node was recovered was recorded as the edge strength.

**Survival score derivation.** The survival node was modeled as a continuous Gaussian variable conditional on its identified parent pathways, yielding a linear conditional probability distribution (CPD) of the form:

E[Survival score | parents] = β_0_ + Σ β_i_ · x_i_

where β_0_ is the intercept (representing the expected survival score when all parent pathway activity scores equal zero, i.e., at the cohort mean given combined Z-score normalization), and β_i_ are partial regression coefficients estimated by maximum likelihood.

Score values exceeding the intercept are theoretically possible when net pathway contributions are positive; the score should not be interpreted as a probability but as a continuous prognostic index calibrated against survival.

### 2.5. Statistical Analysis

Continuous variables were compared using the Mann–Whitney U or Student’s t-test as appropriate. Categorical associations were assessed by Fisher’s exact test. Survival was estimated by the Kaplan–Meier method and compared between groups by the log-rank test. The continuous risk score was applied to each cohort without modification to the model structure or coefficients. For the purpose of risk group visualization, cohort-specific optimal dichotomization thresholds were determined independently within each cohort using maximally selected rank statistics; the prognostic inference is therefore grounded in the continuous score, not in any single cut-point. Univariable and multivariable Cox proportional hazards regressions estimated hazard ratios (HRs) and 95% confidence intervals; covariates entering multivariable models were selected on the basis of univariable significance. Multiple-testing correction used the Benjamini–Hochberg procedure [26]. Two-sided *p* < 0.05 defined statistical significance. Analyses were performed in R v4.1.1 and GraphPad Prism v9. Code is available at https://github.com/chungkok/bnms (accessed 17 June 2023).

## 3. Results

### 3.1. A Bayesian Network Identifies Five Pathways with Direct Conditional Dependence on Myeloma Survival

**Figure 1:**
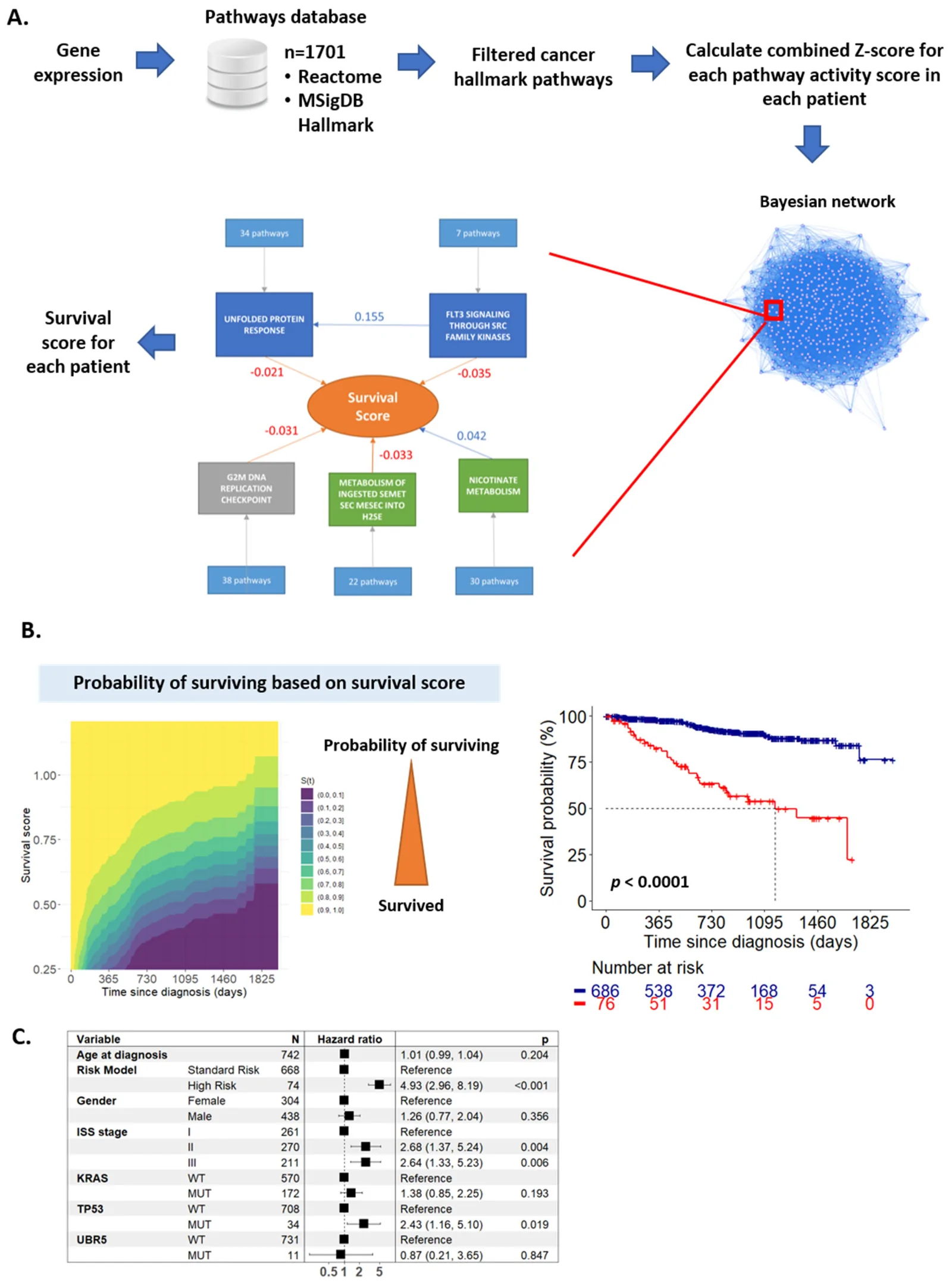
**Development of a pathway-based Bayesian network survival model.** (**A**) Analytical workflow. Gene expression profiles from 762 newly diagnosed MMRF CoMMpass patients were mapped to curated pathway databases (Reactome, MSigDB Hallmark; *n* = 1701 pathways), filtered for cancer-relevant and non-redundant pathways, scored per patient via combined Z-score ssGSEA, and integrated through a Bayesian network. The simplified subnetwork shows the five pathways with direct edges to the survival node and their associated coefficients. (**B**) Kaplan–Meier survival by model-derived risk group in the discovery cohort (log-rank *p* < 0.0001). The left panel illustrates the continuous relationship between survival score and survival probability over time. (**C**) Multivariable Cox proportional hazards analysis of the risk model alongside age, sex, ISS stage, and *KRAS*, *TP53*, *UBR5* mutational status.

Following pathway curation, redundancy filtering, and per-sample ssGSEA scoring (Figure 1A), Bayesian network structure learning identified five pathways with direct edges to the survival node: HALLMARK unfolded protein response (UPR), Reactome FLT3 signaling through SRC family kinases, Reactome G2M DNA replication checkpoint, Reactome metabolism of ingested SeMet/SeC into H2Se (selenium compound metabolism), and Reactome nicotinate metabolism. For the discovery cohort, the fitted Gaussian conditional probability distribution CPD yielded:

Survival score = 0.89 − 0.021(UPR) − 0.035(SRC) − 0.031(G2M) − 0.033(SeMet) + 0.042(Nicotinate)

The intercept of 0.89 reflects the baseline expected survival score at mean pathway activity in the CoMMpass discovery cohort. The coefficient signs indicate the direction of the conditional dependency: negative coefficients indicate that higher activity of UPR, SRC-family signaling, G2M checkpoint, and SeMet metabolism was associated with a lower expected survival score, whereas the positive coefficient on nicotinate metabolism indicates a higher expected survival score. Consistent with these model-derived coefficients, higher activity of UPR, SRC-family signaling, G2M checkpoint, and SeMet metabolism was associated with poorer survival, whereas higher nicotinate metabolism was associated with improved survival.

Applying the optimal score cut-point of 0.77 (maximally selected rank statistic), patients in the discovery cohort were stratified into high-risk (*n* = 76; 10%) and standard-risk (*n* = 686; 90%) groups, with substantial divergence in survival (median 1170 days [95% CI 782–NR] vs. not reached; log-rank *p* < 0.0001; Figure 1B). Bootstrap resampling (R = 100) demonstrated variable stability across the five reported edges: nicotinate metabolism was recovered in 59.6% of resamples, G2M checkpoint in 43.8%, selenium compound metabolism in 37.4%, FLT3/SRC family signaling in 33.1%, and UPR in 9.5% (Table S3). These results indicate that the stability of individual pathway-survival edges varied under resampling. However, the composite survival score, which integrates all five pathway activities, was independently evaluated across the external cohorts.

### 3.2. The Risk Score Is an Independent Factor of Overall Survival

Univariable analysis identified male sex (*p* = 0.019) and advanced ISS stage (*p* < 0.001) as significantly associated with high-risk classification (Table S4). Among recurrently mutated genes, *KRAS* (35.5% vs. 21.7%; *p* = 0.01), *TP53* (19.7% vs. 2.9%; *p* < 0.001), and *UBR5* (5.3% vs. 1.0%; *p* = 0.015) were enriched in the high-risk group (Figure S1 and Table S5). These genes are established cancer driver genes under the COSMIC Cancer Gene Census [27], with *TP53* loss representing a canonical high-risk lesion in MM.

In multivariable Cox regression incorporating age, sex, ISS, and the three mutated genes (*n* = 742 patients with complete covariate data; 20 patients excluded for missing covariate data; 74 high-risk, 668 standard-risk), the risk model retained the strongest independent association with survival (HR 4.93; 95% CI 2.96–8.19; *p* < 0.001), alongside ISS stage II (HR 2.68; 95% CI 1.37–5.24; *p* = 0.004), ISS stage III (HR 2.64; 95% CI 1.33–5.23; *p* = 0.006; overlapping confidence intervals with stage II), and *TP53* mutation (HR 2.43; 95% CI 1.16–5.10; *p* = 0.019) (Figure 1C). Notably, *KRAS* and *UBR5* mutations were not independently prognostic once the pathway-based score was entered, suggesting that the model captures the downstream functional consequences of these driver events.

### 3.3. External Validation Across Five Independent Diagnostic Cohorts

**Figure 2:**
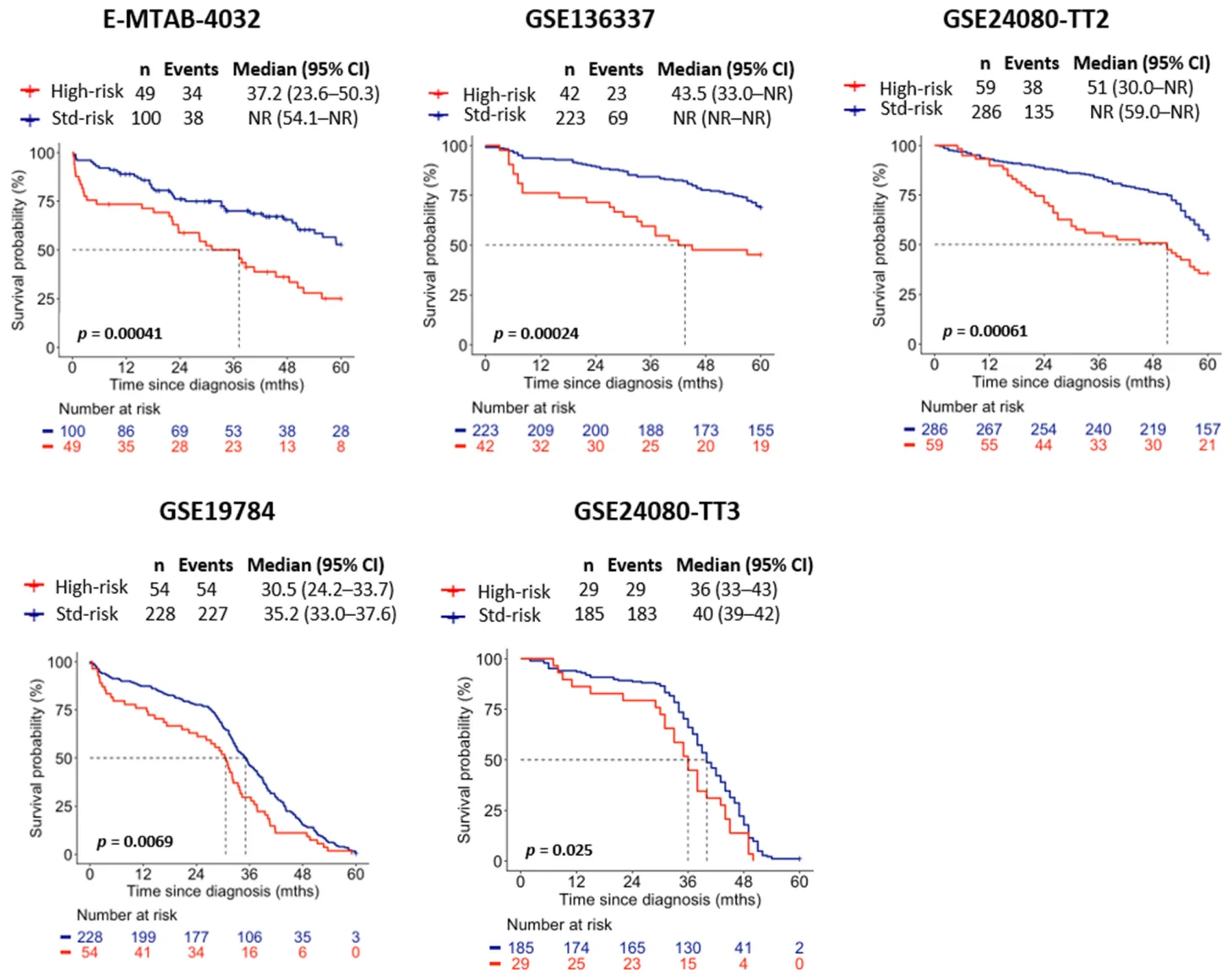
External validation in five independent diagnostic cohorts. Kaplan–Meier survival curves stratified by model-derived risk group across E-MTAB-4032, GSE136337, GSE24080-TT2, GSE19784, and GSE24080-TT3. Median survival, numbers at risk, and log-rank *p* values are shown for each cohort. The dotted line represents 50% survival probability.

The continuous risk score model was applied without modification to 1255 patients across five independent diagnostic cohorts (Figure 2). In each, high-risk patients exhibited significantly inferior survival compared with standard-risk counterparts: E-MTAB-4032 (median 37.2 months vs. NR; *p* = 0.00041); GSE136337 (43.5 months vs. NR; *p* = 0.00024); GSE24080-TT2 (51 months vs. NR; *p* = 0.00061); GSE19784 (30.5 vs. 35.2 months; *p* = 0.0069); and GSE24080-TT3 (36 vs. 40 months; *p* = 0.025). Across cohorts, the model classified an average of 19.7 ± 7.6% of patients as high-risk (range 13.6–32.9%; the higher proportion in E-MTAB-4032 may reflect cohort-specific enrichment for poor-prognosis features), broadly consistent with established estimates of high-risk MM prevalence.

### 3.4. Association Between Cytogenetic Risk and the Risk Score Model

Based on R-ISS and R2-ISS criteria, high-risk cytogenetic abnormalities were defined as del(17p), t(4;14), t(14;16), and gain or amplification of 1q (1q+). We then examined whether our risk model was associated with these abnormalities in external validation cohorts. Among the five independent datasets, only two (E-MTAB-4032 and GSE136337) included cytogenetic information derived from FISH and/or karyotyping.

In E-MTAB-4032 (*n* = 149), there were no statistically significant differences in the distribution of high-risk cytogenetic abnormalities between the standard- and high-risk groups after Benjamini–Hochberg (BH) correction, including del(17p) (BH-adjusted *p* = 1.00), t(4;14) (BH-adjusted *p* = 0.11), and 1q+ (BH-adjusted *p* = 0.081) (Table S6). A similar pattern was observed in GSE136337 (*n* = 265), where none of the assessed cytogenetic features, including del(17p) (BH-adjusted *p* = 0.91), t(4;14) (BH-adjusted *p* = 0.79), t(14;16) (BH-adjusted *p* = 1.00), or 1q+ (BH-adjusted *p* = 0.33), differed significantly between risk groups (Table S7).

Overall, these findings suggest that the risk model may capture prognostic information beyond known high-risk cytogenetic abnormalities in the two cohorts with available FISH data. However, given the modest sample sizes of these subsets, the absence of a statistically significant association should be interpreted cautiously, as it may reflect limited statistical power.

### 3.5. Dynamic Risk Tracking in Post-Treatment and Relapsed/Refractory Disease

To assess longitudinal applicability, we evaluated the model in patients with serial pre- and post-treatment time points, previously treated disease, and relapsed/refractory disease.

**Figure 3:**
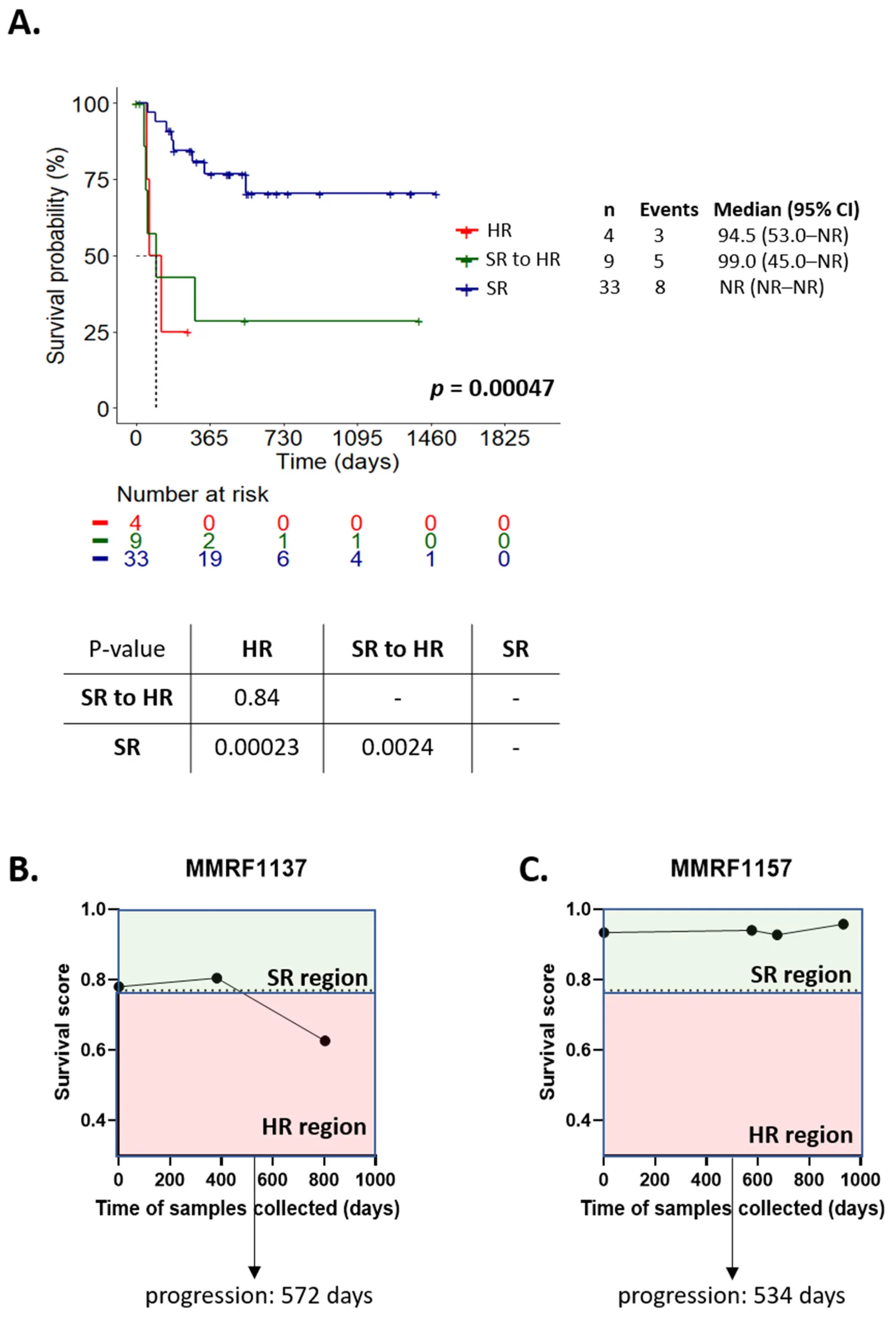
(**A**) Longitudinal risk stratification across serial samples in MMRF patients with paired diagnosis–treatment time points, comparing stable standard-risk (SR; *n* = 33), stable high-risk (HR; *n* = 4), and patients transitioning from standard- to high-risk (SR-to-HR; *n* = 9) (overall log-rank *p* = 0.00047). Pairwise comparisons demonstrate that SR-to-HR patients exhibit survival indistinguishable from stable HR (*p* = 0.84) and significantly inferior to stable SR (*p* = 0.0024). Examples of two cases, MMRF1137 (SR-to-HR) (**B**) and MMRF1157 (SR) (**C**), with patient samples from at least three follow-up time points, were subjected to our model to assess the risk score for each time point. The dotted line refers to predefined risk-score cutoff for the standard-risk (SR; green) and high-risk (HR; red) regions.

In the 46 MMRF patients with longitudinal sampling, post-treatment risk classification stratified survival (Figure 3A). Importantly, patients transitioning from standard-risk at diagnosis to high-risk after treatment (SR-to-HR; *n* = 9) exhibited significantly inferior survival relative to those remaining standard-risk (*n* = 33; median 99.0 vs. NR days; *p* = 0.0024; Figure 3A) and showed no statistically significant difference in survival from patients who were high-risk throughout (*n* = 4; *p* = 0.84; Figure 3A). Examples of cases in which patients with at least three follow-up time points available are shown in Figure 3B,C. Although these subgroups are small and the finding is therefore exploratory, the transition was driven primarily by post-treatment elevation in three of the five model pathways (G2M checkpoint *p* = 0.006; SeMet metabolism *p* = 0.009; SRC-family signaling *p* = 0.047; Figure S2), consistent with treatment-induced selection of proliferative and stress-response programs. These findings suggest that the risk model can be used dynamically to monitor patient journeys.

**Figure 4:**
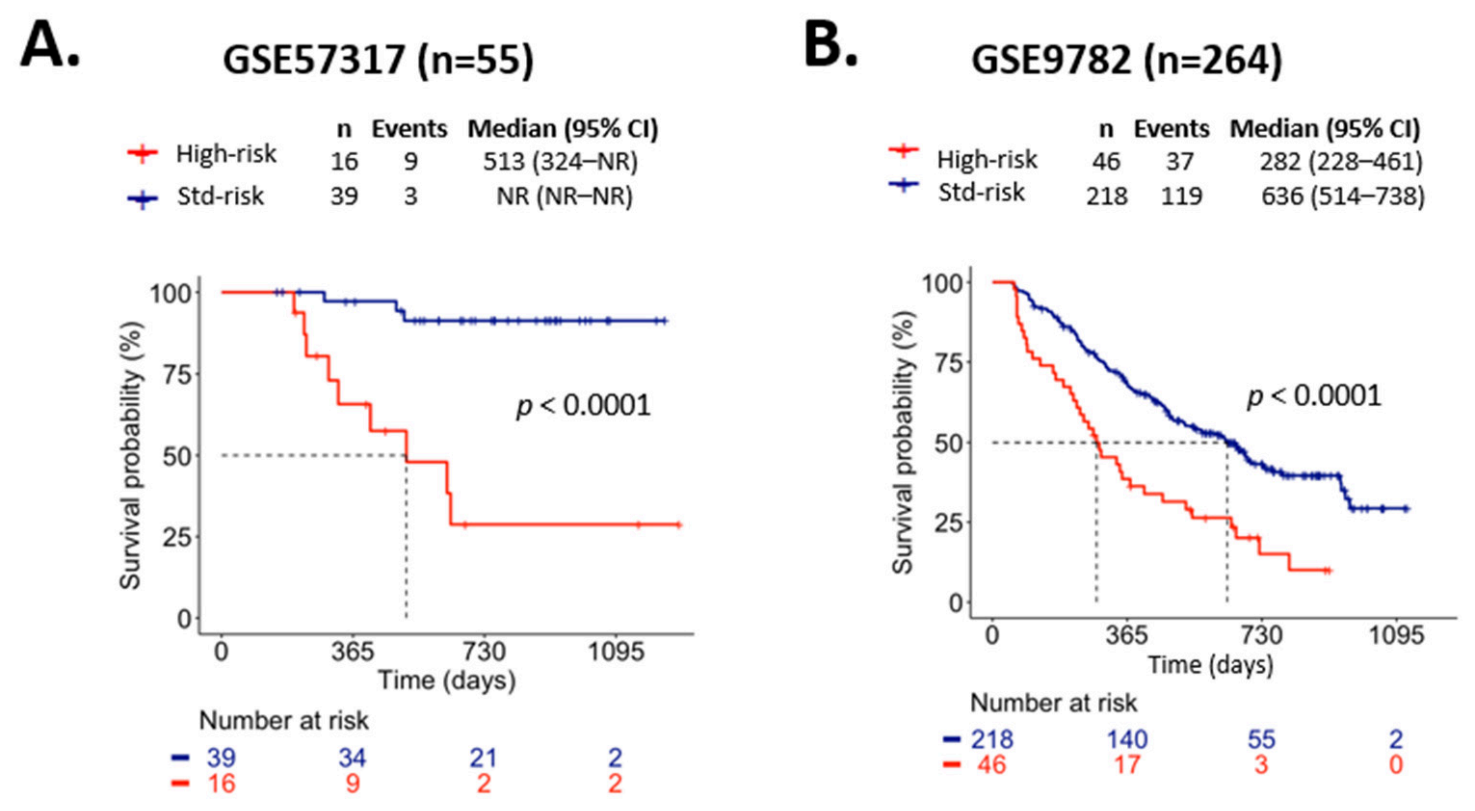
Validation in post-treatment and relapsed/refractory disease. Kaplan–Meier survival curves for (**A**) previously treated (GSE57317; *n* = 55) and (**B**) relapsed/refractory (GSE9782; *n* = 264) cohorts, stratified by model-derived risk group (log-rank *p* < 0.0001 for both). The dotted line represents 50% survival probability.

In an independent post-treatment cohort (GSE57317; *n* = 55), 16 patients (29%) were classified as high-risk and exhibited markedly shorter survival (median 513 days vs. NR; *p* < 0.0001; Figure 4A). In a relapsed/refractory cohort treated with bortezomib (GSE9782; *n* = 264), 46 patients (17%) were classified as high-risk with significantly inferior survival (median 282 vs. 636 days; *p* < 0.0001; Figure 4B). Collectively, these findings establish that the model retains discriminative prognostic value across multiple disease states and treatment eras.

**Figure 5:**
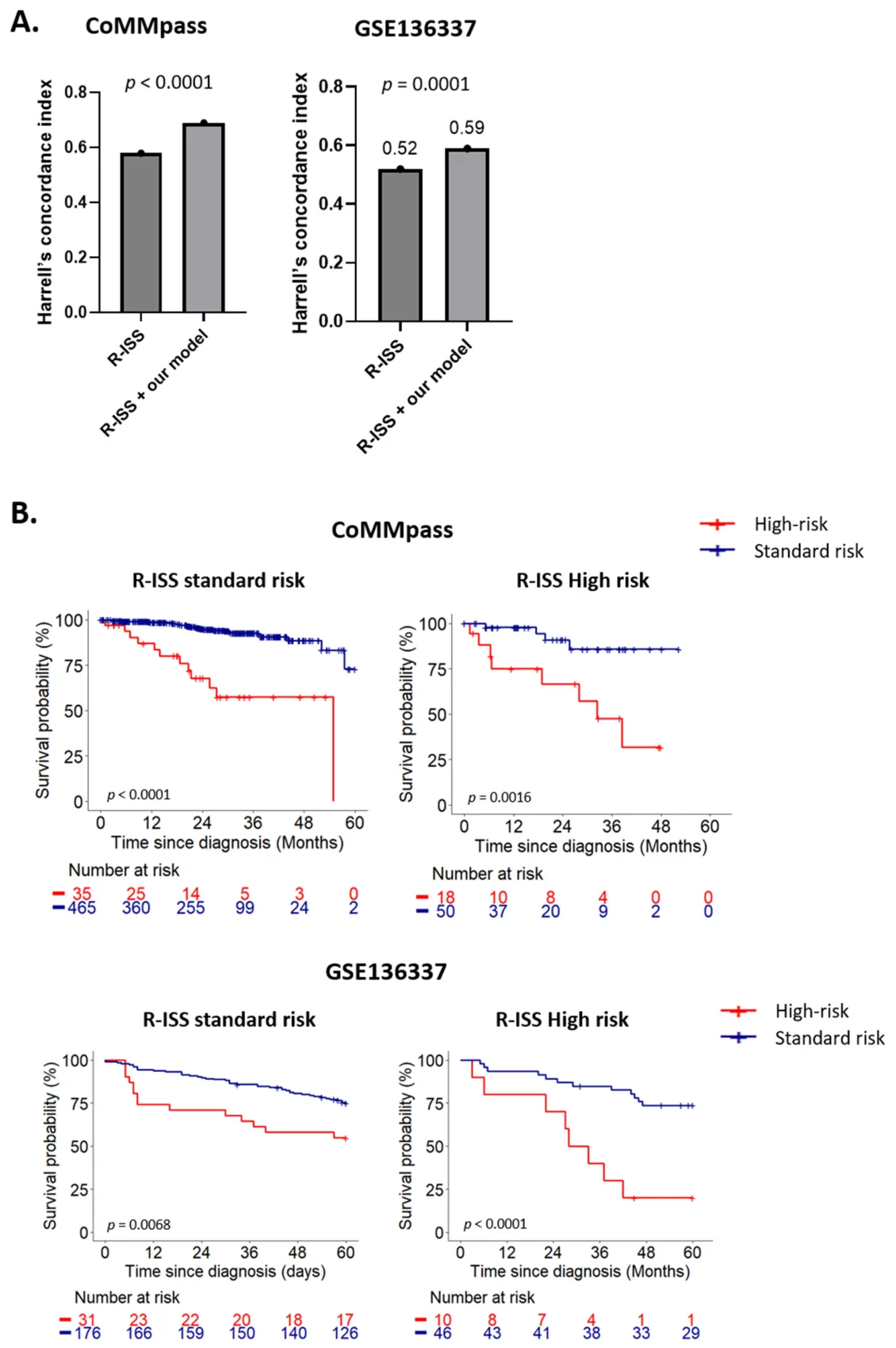
**Clinical value of our risk score beyond R-ISS.** (**A**) Harrell’s concordance index for models incorporating R-ISS alone or R-ISS plus our proposed risk score in the CoMMpass and GSE136337 cohorts. *p* values were calculated using likelihood-ratio tests comparing the nested models. (**B**) Kaplan–Meier survival curves stratified by our proposed risk score within R-ISS-defined standard-risk and high-risk subgroups in the CoMMpass and GSE136337 cohorts. Numbers at risk and log-rank *p* values are shown for each subgroup.

To assess whether the score provides prognostic information beyond established staging, we fitted a Cox proportional hazards model for overall survival containing R-ISS alone or R-ISS plus the proposed score. Addition of the score significantly improved model discrimination in both cohorts (likelihood-ratio test: CoMMpass, *p* < 0.0001; GSE136337, *p* = 0.001), with an increase in Harrell’s C-index from 0.58 to0.69 in CoMMpass and from 0.52 to 0.59 in GSE136337 (Figure 5A).

To determine whether the score identifies prognostic heterogeneity within conventionally defined risk groups, Kaplan–Meier survival analyses were performed within R-ISS-defined standard- and high-risk subgroups in two independent cohorts. The score significantly stratified survival among R-ISS standard-risk patients in CoMMpass (*p* < 0.0001) and GSE136337 (*p* = 0.0068) (Figure 5B). Significant survival separation was also observed within the R-ISS high-risk subgroups in both cohorts.

## 4. Discussion

We present a Bayesian network-derived pathway risk model that stratifies MM survival reproducibly across diagnostic, post-treatment, and relapsed/refractory settings. The model offers three distinguishing features relative to incumbent classifiers. First, it is parsimonious and biologically interpretable: five pathways with direct conditional dependence on survival recapitulate canonical MM biology, consistent with prior work showing that a small number of genes or pathways can carry much of the discriminatory power of larger expression-based risk signatures [28]. UPR activation reflects the proteostatic burden of immunoglobulin-secreting plasma cells and is implicated in proteasome inhibitor sensitivity [29,30], and expression-based signatures have similarly been associated with outcome in patients treated with the proteasome inhibitor bortezomib [31]. SRC-family kinase signaling downstream of FLT3 has been implicated in MM cell survival and microenvironmental cross-talk [32,33,34,35], and G2M checkpoint activity captures proliferative drive [22,36]. The metabolic axes identified by the model, namely selenium and nicotinate/NAD+ metabolism, point to redox and energy metabolism vulnerabilities increasingly recognized as therapeutic frontiers in hematological malignancies [37,38]. The mechanistic evidence supporting the five identified pathways is not uniform. UPR activation and G2M checkpoint activity have established links to myeloma biology, including proteasome inhibitor response and proliferative activity, respectively. In contrast, the associations involving FLT3/SRC, selenium metabolism, and nicotinate metabolism are less well established in myeloma and should be regarded as hypothesis-generating. Functional studies will be required to determine whether these pathways have a direct role in myeloma disease progression or treatment response.

Second, the risk model retains its strong prognostic association with survival after adjustment for variables such as ISS stage and *TP53* mutation in multivariable Cox regression. Both variables remain independently prognostic in the same model, indicating that the score adds non-redundant information rather than substituting for established clinical and genomic markers. The model also does not significantly correlate with FISH-defined high-risk cytogenetics in the two independent cohorts where these were tested. Together, these findings suggest the score captures a transcriptional axis of risk that is complementary to chromosomal lesions and clinical staging, with potential to refine R-ISS-based stratification.

Third, and most consequential for translational application, the model retains prognostic validity when applied serially. Patients who transitioned from SR- to HR-status following treatment did not have significantly different survival outcomes from those with stable HR-status. However, the stable HR group comprised only four patients, limiting statistical power and precluding a robust assessment of equivalence. Nonetheless, the observed SR-to-HR transition identifies a population that may warrant closer clinical surveillance or consideration for clinical trial enrollment. This dynamic risk stratification addresses a current gap in practice, where R-ISS is not routinely re-evaluated post-induction, and identifies a population that may warrant closer surveillance or consideration for trial enrollment; whether score-guided intervention improves outcomes requires prospective testing.

Several limitations should be acknowledged. The discovery and the majority of validation cohorts are derived from CD138-selected bulk RNA-sequencing or microarray platforms; cross-platform applicability requires further methodological harmonization. Treatment regimens differ across cohorts and eras, which may confound post-treatment analyses. The serial MMRF cohort, while informative, is modest in sample size. Thus, the dynamic-tracking finding should be regarded as exploratory and warrants confirmation in larger longitudinal series. Finally, all analyses are retrospective; prospective evaluation within an IMWG-aligned clinical trial framework is the necessary next step.

The validation cohorts span induction strategies from conventional VAD- to CAD-based chemotherapy (GSE24080-TT2) through bortezomib- and lenalidomide-containing triplet regimens (GSE24080-TT3, GSE57317) (Tables S1 and S2), and the score retained significant prognostic discrimination across treatment backgrounds. However, none of the available cohorts included patients treated with daratumumab or other CD38-targeted quadruplet regimens. Our findings should therefore be interpreted as evidence of prognostic value. Formal evaluation of predictive value will require treatment-stratified cohorts with appropriate treatment-response data and assessment of a score-by-treatment interaction.

Because serial samples in this dataset were collected at clinically defined time points, including suspected complete response, recurrence, or disease progression [21], rather than according to a prespecified longitudinal surveillance schedule, the temporal relationship between molecular risk stratification and subsequent disease progression could not be determined. Accordingly, the longitudinal analysis demonstrates the feasibility of dynamic risk reassessment following treatment but does not establish whether molecular risk classification provides clinically meaningful lead time over conventional measures of disease progression. Prospective studies incorporating protocol-defined serial sampling independent of clinical progression are required to determine the temporal and clinical utility of molecular risk reclassification.

None of the cohorts used in this study included MRD measurements or sufficiently structured longitudinal treatment-response data. Therefore, whether the score predicts MRD persistence, early relapse, or frontline treatment resistance remains unknown. Prospective validation incorporating MRD, treatment response, and serial transcriptomic profiling will be required to determine whether the score provides clinically actionable information beyond conventional risk stratification.

Although transcriptomic profiling may currently present a practical consideration for clinical implementation, RNA sequencing/whole-transcriptome sequencing (WTS) is increasingly feasible within molecular diagnostic workflows. Its application has been demonstrated in MM and, more recently, in prospective hematological diagnostics, supporting integration into routine clinical practice [39,40,41,42]. Our pipeline could be incorporated into this workflow as an automated downstream analysis of clinically generated RNA-seq data, producing a standardized risk score for inclusion in the molecular pathology report alongside other established biomarkers. Although the current implementation uses whole-transcriptome sequencing, the five-pathway signature may ultimately be translated into a targeted gene-expression assay, similar to other clinically deployed expression-based tests. Such an approach would require independent assay development and prospective analytical and clinical validation.

## 5. Conclusions

This pathway-based Bayesian network constitutes a reproducible, biologically grounded, and dynamically applicable risk model for multiple myeloma. Subject to prospective validation, it offers a route to longitudinal risk stratification that complements existing clinical and cytogenetic frameworks and may support enrichment strategies for high-risk patients in clinical trials.

## Data Availability

All datasets analyzed are publicly available from ArrayExpress (E-MTAB-4032), Gene Expression Omnibus (GSE136337, GSE19784, GSE24080, GSE57317, GSE9782), and the MMRF Researcher Gateway (https://research.themmrf.org and www.themmrf.org) and was accessed on 2 June 2025. Data were retrieved from the Genomic Data Commons (GDC) data portal on 9 January 2021 (https://portal.gdc.cancer.gov/). Risk model code and scripts are available at https://github.com/chungkok/bnms and was accessed 17 June 2023.

## Supplementary Materials

The following supporting information can be downloaded at: https://www.mdpi.com/article/10.3390/cancers18172864/s1, Figure S1: Somatic mutation enrichment in high-risk patients; Figure S2: Paired pathway activity scores at diagnosis vs. post-treatment for the five model pathways; Table S1: Independent diagnostic validation cohorts; Table S2: Cohorts for previously treated and relapsed/refractory analyses; Table S3: Bootstrap edge stability for the five pathways with direct edges to the survival node; Table S4: Clinical characteristics of high-risk vs. standard-risk patients; Table S5: Somatic mutations associated with standard-risk vs. high-risk patients; Table S6: Distribution of high-risk cytogenetic abnormalities in the E-MTAB-4032 cohort stratified by risk score group; Table S7: Distribution of high-risk cytogenetic abnormalities in the GSE136337 cohort stratified by risk score group.

## References

[1] Mafra A., Laversanne M., Marcos-Gragera R., Chaves H.V.S., McShane C., Bray F., Znaor A. (2025). The global multiple myeloma incidence and mortality burden in 2022 and predictions for 2045. J. Natl. Cancer Inst..

[2] Turesson I., Bjorkholm M., Blimark C.H., Kristinsson S., Velez R., Landgren O. (2018). Rapidly changing myeloma epidemiology in the general population: Increased incidence, older patients, and longer survival. Eur. J. Haematol..

[3] Palumbo A., Avet-Loiseau H., Oliva S., Lokhorst H.M., Goldschmidt H., Rosinol L., Richardson P., Caltagirone S., Lahuerta J.J., Facon T. (2015). Revised International Staging System for Multiple Myeloma: A Report From International Myeloma Working Group. J. Clin. Oncol..

[4] Vo J.N., Wu Y.M., Mishler J., Hall S., Mannan R., Wang L., Ning Y., Zhou J., Hopkins A.C., Estill J.C. (2022). The genetic heterogeneity and drug resistance mechanisms of relapsed refractory multiple myeloma. Nat. Commun..

[5] van Laar R., Flinchum R., Brown N., Ramsey J., Riccitelli S., Heuck C., Barlogie B., Shaughnessy J.D. (2014). Translating a gene expression signature for multiple myeloma prognosis into a robust high-throughput assay for clinical use. BMC Med. Genom..

[6] Kuiper R., Broyl A., de Knegt Y., van Vliet M.H., van Beers E.H., van der Holt B., el Jarari L., Mulligan G., Gregory W., Morgan G. (2012). A gene expression signature for high-risk multiple myeloma. Leukemia.

[7] Ebert L.M., Vandyke K., Johan M.Z., DeNichilo M., Tan L.Y., Myo Min K.K., Weimann B.M., Ebert B.W., Pitson S.M., Zannettino A.C.W. (2022). Desmoglein-2 expression is an independent predictor of poor prognosis patients with multiple myeloma. Mol. Oncol..

[8] McClure B.J., Toomes J., Best C., Yong G., Lee A., Bonder C., Kok C. (2026). Elevated desmoglein-2 expression in multiple myeloma is a prognostic marker across genomic subtypes with impact on high-risk cytogenetics and a distinct gene expression profile. Br. J. Haematol..

[9] Mason M.J., Schinke C., Eng C.L.P., Towfic F., Gruber F., Dervan A., White B.S., Pratapa A., Guan Y., Chen H. (2020). Multiple Myeloma DREAM Challenge reveals epigenetic regulator PHF19 as marker of aggressive disease. Leukemia.

[10] Lee C.H., Zhang Y., McClure B.J., Yong A., Scott H.S., Kok C.H. (2026). Shaping the future of multiple myeloma with artificial intelligence and digital twins: From concept to clinic. Front. Digit. Health.

[11] Scutari M. (2010). Learning Bayesian Networks with the bnlearn R Package. J. Stat. Softw..

[12] Sondka Z., Bamford S., Cole C.G., Ward S.A., Dunham I., Forbes S.A. (2018). The COSMIC Cancer Gene Census: Describing genetic dysfunction across all human cancers. Nat. Rev. Cancer.

[13] Skerget S., Penaherrera D., Chari A., Jagannath S., Siegel D.S., Vij R., Orloff G., Kakubowaik A., Niesvizky R., Liles D. (2024). Comprehensive molecular profiling of multiple myeloma identifies refined copy number and expression subtypes. Nat. Genet..

[14] Danziger S.A., McConnell M., Gockley J., Young M.H., Rosenthal A., Schmitz F., Reiss D.J., Farmer P., Alapat D.V., Singh A. (2020). Bone marrow microenvironments that contribute to patient outcomes in newly diagnosed multiple myeloma: A cohort study of patients in the Total Therapy clinical trials. PLoS Med..

[15] Kryukov F., Nemec P., Radova L., Kryukova E., Okubote S., Minarik J., Stefanikova Z., Pour L., Hajek R. (2016). Centrosome associated genes pattern for risk sub-stratification in multiple myeloma. J. Transl. Med..

[16] Broyl A., Hose D., Lokhorst H., de Knegt Y., Peeters J., Jauch A., Bertsch U., Buijs A., Stevens-Kroef M., Beverloo H.B. (2010). Gene expression profiling for molecular classification of multiple myeloma in newly diagnosed patients. Blood.

[17] Barlogie B., Tricot G., Rasmussen E., Anaissie E., van Rhee F., Zangari M., Fassas A., Hollmig K., Pineda-Roman M., Shaughnessy J. (2006). Total therapy 2 without thalidomide in comparison with total therapy 1: Role of intensified induction and posttransplantation consolidation therapies. Blood.

[18] Mitchell J.S., Li N., Weinhold N., Forsti A., Ali M., van Duin M., Thorleifsson G., Johnson D.C., Chen B., Halvarsson B.M. (2016). Genome-wide association study identifies multiple susceptibility loci for multiple myeloma. Nat. Commun..

[19] Zhan F., Huang Y., Colla S., Stewart J.P., Hanamura I., Gupta S., Epstein J., Yaccoby S., Sawyer J., Burington B. (2006). The molecular classification of multiple myeloma. Blood.

[20] van Rhee F., Szymonifka J., Anaissie E., Nair B., Waheed S., Alsayed Y., Petty N., Shaughnessy J.D., Hoering A., Crowley J. (2010). Total Therapy 3 for multiple myeloma: Prognostic implications of cumulative dosing and premature discontinuation of VTD maintenance components, bortezomib, thalidomide, and dexamethasone, relevant to all phases of therapy. Blood.

[21] Keats J.J., Craig D.W., Liang W., Venkata Y., Kurdoglu A., Aldrich J., Auclair D., Allen K., Harrison B., Jewell S. (2013). Interim Analysis Of The Mmrf Commpass Trial, a Longitudinal Study In Multiple Myeloma Relating Clinical Outcomes To Genomic and Immunophenotypic Profiles. Blood.

[22] Liberzon A., Birger C., Thorvaldsdottir H., Ghandi M., Mesirov J.P., Tamayo P. (2015). The Molecular Signatures Database (MSigDB) hallmark gene set collection. Cell Syst..

[23] Gillespie M., Jassal B., Stephan R., Milacic M., Rothfels K., Senff-Ribeiro A., Griss J., Sevilla C., Matthews L., Gong C. (2022). The reactome pathway knowledgebase 2022. Nucleic Acids Res..

[24] Carenzo A., Pistore F., Serafini M.S., Lenoci D., Licata A.G., De Cecco L. (2022). Hacksig: A unified and tidy R framework to easily compute gene expression signature scores. Bioinformatics.

[25] Lee E., Chuang H.Y., Kim J.W., Ideker T., Lee D. (2008). Inferring pathway activity toward precise disease classification. PLoS Comput. Biol..

[26] Benjamini Y. (1995). Controlling the false discovery rate: A practical and powerful approach to multiple testing. J. R Stat. Soc. Ser. B.

[27] Vogelstein B., Papadopoulos N., Velculescu V.E., Zhou S., Diaz L.A., Kinzler K.W. (2013). Cancer genome landscapes. Science.

[28] Heuck C.J., Qu P., van Rhee F., Waheed S., Usmani S.Z., Epstein J., Zhang Q., Edmondson R., Hoering A., Crowley J. (2014). Five gene probes carry most of the discriminatory power of the 70-gene risk model in multiple myeloma. Leukemia.

[29] Iwakoshi N.N., Lee A.H., Vallabhajosyula P., Otipoby K.L., Rajewsky K., Glimcher L.H. (2003). Plasma cell differentiation and the unfolded protein response intersect at the transcription factor XBP-1. Nat. Immunol..

[30] Meister S., Schubert U., Neubert K., Herrmann K., Burger R., Gramatzki M., Hahn S., Schreiber S., Wilhelm S., Herrmann M. (2007). Extensive immunoglobulin production sensitizes myeloma cells for proteasome inhibition. Cancer Res..

[31] Mulligan G., Mitsiades C., Bryant B., Zhan F., Chng W.J., Roels S., Koenig E., Fergus A., Huang Y., Richardson P. (2007). Gene expression profiling and correlation with outcome in clinical trials of the proteasome inhibitor bortezomib. Blood.

[32] Ho M., Xiao A., Yi D., Zanwar S., Bianchi G. (2022). Treating Multiple Myeloma in the Context of the Bone Marrow Microenvironment. Curr. Oncol..

[33] Kazi J.U., Ronnstrand L. (2019). The role of SRC family kinases in FLT3 signaling. Int. J. Biochem. Cell Biol..

[34] Robinson L.J., Xue J., Corey S.J. (2005). Src family tyrosine kinases are activated by Flt3 and are involved in the proliferative effects of leukemia-associated Flt3 mutations. Exp. Hematol..

[35] Steiner N., Johrer K., Plewan S., Brunner-Veber A., Gobel G., Nachbaur D., Wolf D., Gunsilius E., Untergasser G. (2020). The FMS like Tyrosine Kinase 3 (FLT3) Is Overexpressed in a Subgroup of Multiple Myeloma Patients with Inferior Prognosis. Cancers.

[36] Oshi M., Takahashi H., Tokumaru Y., Yan L., Rashid O.M., Matsuyama R., Endo I., Takabe K. (2020). G2M Cell Cycle Pathway Score as a Prognostic Biomarker of Metastasis in Estrogen Receptor (ER)-Positive Breast Cancer. Int. J. Mol. Sci..

[37] Mitchell S.R., Larkin K., Grieselhuber N.R., Lai T.H., Cannon M., Orwick S., Sharma P., Asemelash Y., Zhang P., Goettl V.M. (2019). Selective targeting of NAMPT by KPT-9274 in acute myeloid leukemia. Blood Adv..

[38] Soncini D., Becherini P., Ladisa F., Ravera S., Chedere A., Gelli E., Giorgetti G., Martinuzzi C., Piacente F., Mastracci L. (2025). NAD+ metabolism restriction boosts high-dose melphalan efficacy in patients with multiple myeloma. Blood Adv..

[39] Emde-Rajaratnam M., Beck S., Benes V., Salwender H., Bertsch U., Scheid C., Hanel M., Weisel K., Hielscher T., Raab M.S. (2023). RNA-sequencing based first choice of treatment and determination of risk in multiple myeloma. Front. Immunol..

[40] Altburger C., Menzel M., Beck S., Lorenz K., Brygider M.L., Ploeger C., Kahles A., Ball M., Kirchner M., Schnecko F. (2026). Implementing whole genome and transcriptome sequencing for cancer patients in routine healthcare: A comprehensive guide to costing. Br. J. Cancer.

[41] Truger M., Meggendorfer M., Walter W., Hutter S., Alsdorf W., Massenkeil G., Martens U.M., Kern W., Horst K., Kuhn C. (2026). Solving Riddles Through Sequencing (SIRIUS): Unlocking hematologic diagnoses by whole genome and transcriptome sequencing. Leukemia.

[42] Cuppen E., Elemento O., Rosenquist R., Nikic S., IJzerman M., Zaleski I.D., Frederix G., Levin L.A., Mullighan C.G., Buettner R. (2022). Implementation of Whole-Genome and Transcriptome Sequencing Into Clinical Cancer Care. JCO Precis. Oncol..

