# Supplementary material for: Dynamic Prediction of Survival Outcomes in Multiple Myeloma
Source: Cancers (Basel). 2026 Sep 4;18(17):2864. doi: 10.3390/cancers18172864 (PMC13564550; doi:10.3390/cancers18172864)
Supplement: Supplementary file 1 [file cancers-18-02864-s001.zip › cancers-4425748-supplementary.pdf]

Supplementary Materials

Dynamic Prediction of Survival Outcomes in Multiple Myeloma

Kelly Quek[1,*], Cindy H. Lee[2,3], Yang Zhang[3,4,5,6], Barbara J McClure[6], Runzhe Chen[7], Hamish S. Scott[4,5,6], Kate Vandyke[5,8], Andrew C.W. Zannettino[5,8] and Chung Hoow Kok[3,4,5,6,*]

1. Accenture, Singapore
2. Department of Haematology, Royal Adelaide Hospital, Central Adelaide Local Health Network, Adelaide, SA, Australia
3. School of Medicine, College of Health, Adelaide University, Adelaide, SA, Australia
4. Department of Genetics and Molecular Pathology, SA Pathology, Adelaide, SA, Australia
5. School of Pharmacy and Biomedical Science, College of Health, Adelaide University, Adelaide, SA, Australia
6. Centre for Cancer Biology, an alliance between SA Pathology and Adelaide University, College of Health, Adelaide, SA, Australia
7. Department of Health Promotion and Behavioral Sciences, School of Public Health, The University of Texas Health Science Center at Houston, Houston, 77030, TX, USA
8. Precision Cancer Medicine Theme, South Australian Health & Medical Research Institute (SAHMRI), Adelaide, SA, Australia

*Correspondence: kelly.quek@accenture.com (K.Q.); chung.kok@adelaide.edu.au (C.H.K.)

This Supplementary Materials file contains:

Supplementary Figures – 2

Supplementary Tables – 7

References

Supplementary Figures

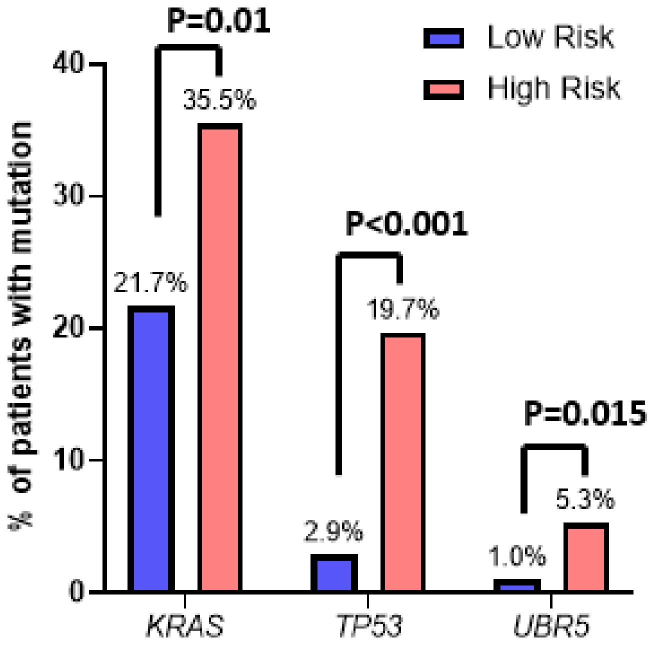

Figure S1. **Somatic mutation enrichment in high-risk patients.** Frequency of *KRAS*, *TP53*, and *UBR5* somatic mutations across high-risk and standard-risk patients in the discovery cohort (Fisher's exact test).

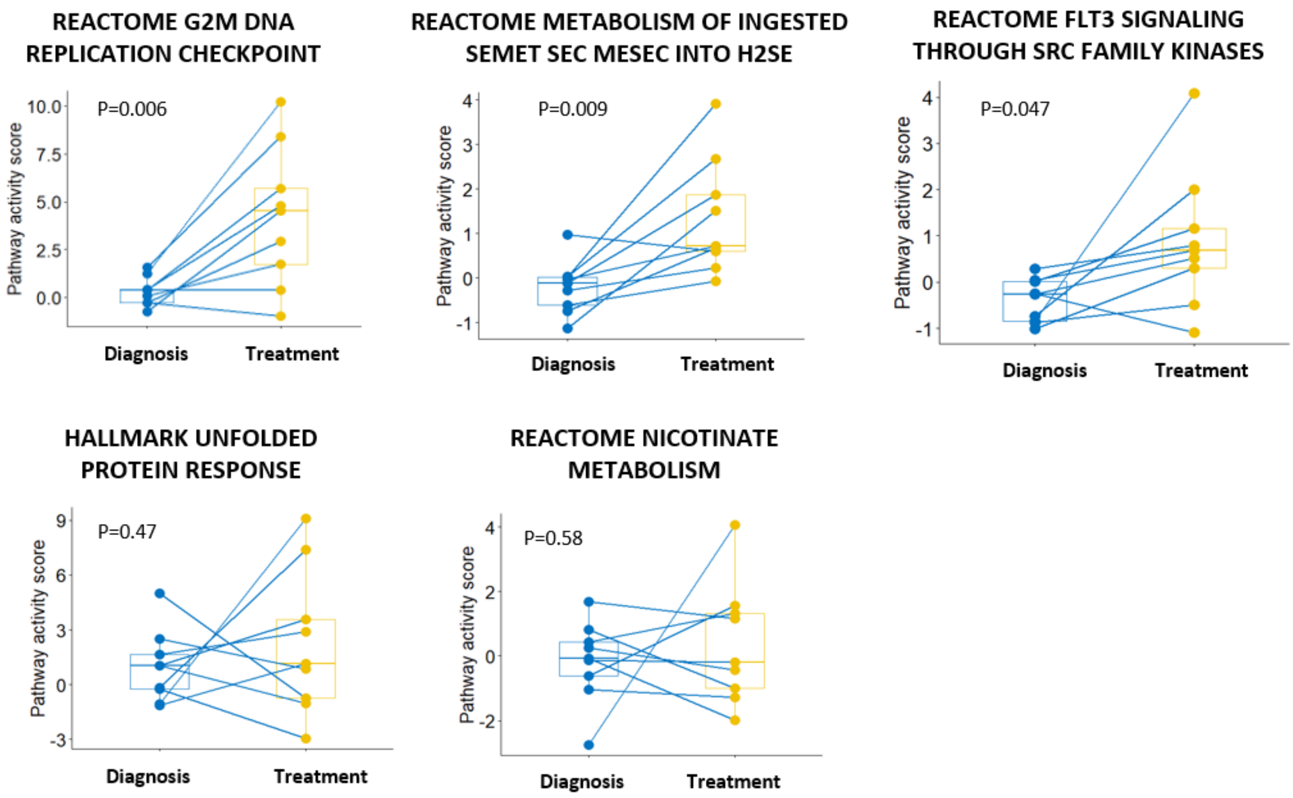

Figure S2. Paired pathway activity scores at diagnosis and post-treatment across the five model-derived pathways. Significant post-treatment increases were observed in G2M DNA replication checkpoint (P = 0.006), selenium compound metabolism (P = 0.009), and FLT3-SRC-family signalling (P = 0.047); UPR (P = 0.47) and nicotinate metabolism (P = 0.58) showed no significant change.

Supplementary Tables

Table S1. Independent diagnostic validation cohorts.

Accession #	Source	n	Cell types	Treatment	Platform
E-MTAB-4032 (ArrayExpress) [1]	University Hospital Brno (CZ); University Hospital Olomouc (CZ); University Hospital Bratislava (SK)	149	CD138-purified BM myeloma plasma cells	Bortezomib-, thalidomide-, lenalidomide-based, or other regimens	Affymetrix GeneChip Human Gene ST 1.0
GSE136337 [2]	Only included Total Therapy 3B (TT3B), TT4 and TT5	265	CD138-purified myeloma plasma cells	Thalidomide, dexamethasone, autologous BMT	Affymetrix HG U133 Plus 2.0
GSE19784 [3]	HOVON-65 GMMG-HD4	282	CD138-purified BM myeloma plasma cells	Vincristine, doxorubicin, and dexamethasone or bortezomib, doxorubicin, and dexamethasone followed by high-dose melphalan and autologous stem-cell transplantation followed by thalidomide or bortezomib maintenance	Affymetrix HG U133 Plus 2.0
GSE24080 (TT2) [4-6]	TT2	345	CD138-purified BM myeloma plasma cells	VAD-DCEP-CAD induction; tandem autotransplant (MEL 200); D-PACE consolidation; interferon maintenance with DEX	Affymetrix HG U133 Plus 2.0
GSE24080 (TT3) [5-7]	TT3	214	CD138-purified BM myeloma plasma cells	2 cycles VTD-PACE (bortezomib, thalidomide, dexamethasone; 4-day infusion of cisplatin, doxorubicin, cyclophosphamide, etoposide)	Affymetrix HG U133 Plus 2.0

Source, sample size, sample type, treatment regimen, and assay platform for the five external diagnostic validation cohorts (total n=1,255).

Table S2. Cohorts for previously treated and relapsed/refractory analyses.

Accession #	Source	n	Cell types	Treatment	Platform
MMRF [8,9]	MMRF CoMMpass	46	CD138-purified BM myeloma plasma cells; previously treated	Various	RNA-sequencing
GSE57317 [10]	Total Therapy 6 (TT6)	55	CD138-purified BM myeloma plasma cells; previously treated	VRD (bortezomib/lenalidomide/dexamethasone) alternating with VMD (bortezomib/melphalan/dexamethasone)	Affymetrix HG U133 Plus 2.0
GSE9782 [11]	Millennium Pharmaceuticals	264	CD138-purified BM myeloma plasma cells; relapsed	Various, all including bortezomib	Affymetrix HG U133A and U133B

Source, sample size, treatment, and platform details for the three cohorts used to evaluate the model in post-treatment and relapsed/refractory disease settings.

Table S3: Bootstrap edge stability for the five pathways with direct edges to the survival node.

**Pathway**	**Edge Strength***
Nicotinate metabolism	0.596
G2M DNA replication checkpoint	0.438
Selenium compound metabolism (SeMet)	0.374
FLT3 signalling through SRC family kinases	0.331
Unfolded protein response (UPR)	0.095

* *Edge strength represents the proportion of bootstrap resamples in which the corresponding directed edge between the pathway node and the survival node was recovered*

Table S4. Clinical characteristic of high-risk vs standard-risk patients.

Variable	Level	Standard-risk	High -risk	p
**n**		686	76	
**Age at diagnosis (mean (SD))**		62.82 (10.44)	61.05 (11.76)	0.168
**OS status (%)**	**Alive**	**632 (92.1)**	**46 (60.5)**	**<0.001**
	**Dead**	**54 (7.9)**	**30 (39.5)**	
**Gender (%)**	**Female**	**290 (42.3)**	**21 (27.6)**	**0.019**
	**Male**	**396 (57.7)**	**55 (72.4)**	
**ISS stage (%)**	**I**	**246 (36.8)**	**15 (20.3)**	**<0.001**
	**II**	**248 (37.1)**	**22 (29.7)**	
	**III**	**174 (26.0)**	**37 (50.0)**	

Table S5. Somatic mutations that associated with standard-risk vs high-risk patients.

Mutation	Overall	Standard Risk	High Risk	p-value
n	762	686	76	
AFF3 (%)	11 (1.4)	10 (1.5)	1 (1.3)	1
AKAP9 (%)	11 (1.4)	9 (1.3)	2 (2.6)	0.683
ARID1A (%)	12 (1.6)	12 (1.7)	0 (0.0)	0.499
ATM (%)	23 (3.0)	22 (3.2)	1 (1.3)	0.575
ATRX (%)	11 (1.4)	10 (1.5)	1 (1.3)	1
BIRC6 (%)	14 (1.8)	13 (1.9)	1 (1.3)	1
BRAF (%)	52 (6.8)	45 (6.6)	7 (9.2)	0.529
CCND1 (%)	12 (1.6)	12 (1.7)	0 (0.0)	0.499
CNTNAP2 (%)	11 (1.4)	9 (1.3)	2 (2.6)	0.683
CREBBP (%)	13 (1.7)	10 (1.5)	3 (3.9)	0.261
CSMD3 (%)	37 (4.9)	34 (5.0)	3 (3.9)	0.915
CTNNA2 (%)	12 (1.6)	10 (1.5)	2 (2.6)	0.768
CYLD (%)	26 (3.4)	25 (3.6)	1 (1.3)	0.467
DCC (%)	13 (1.7)	11 (1.6)	2 (2.6)	0.849
FAM135B (%)	11 (1.4)	9 (1.3)	2 (2.6)	0.683
FAT1 (%)	34 (4.5)	30 (4.4)	4 (5.3)	0.949
FAT3 (%)	37 (4.9)	34 (5.0)	3 (3.9)	0.915
FAT4 (%)	46 (6.0)	41 (6.0)	5 (6.6)	1
FGFR3 (%)	30 (3.9)	27 (3.9)	3 (3.9)	1

FLT4 (%)	12 (1.6)	11 (1.6)	1 (1.3)	1
IRF4 (%)	20 (2.6)	19 (2.8)	1 (1.3)	0.708
KMT2A (%)	13 (1.7)	13 (1.9)	0 (0.0)	0.457
KMT2C (%)	13 (1.7)	13 (1.9)	0 (0.0)	0.457
KMT2D (%)	12 (1.6)	11 (1.6)	1 (1.3)	1
KRAS (%)	**176 (23.1)**	**149 (21.7)**	**27 (35.5)**	**0.01**
LRP1B (%)	42 (5.5)	37 (5.4)	5 (6.6)	0.869
MAX (%)	23 (3.0)	20 (2.9)	3 (3.9)	0.884
MUC16 (%)	66 (8.7)	57 (8.3)	9 (11.8)	0.41
NCOR1 (%)	12 (1.6)	10 (1.5)	2 (2.6)	0.768
NF1 (%)	13 (1.7)	12 (1.7)	1 (1.3)	1
NFKB2 (%)	13 (1.7)	13 (1.9)	0 (0.0)	0.457
NRAS (%)	155 (20.3)	141 (20.6)	14 (18.4)	0.773
PIM1 (%)	12 (1.6)	11 (1.6)	1 (1.3)	1
POLR2A (%)	11 (1.4)	10 (1.5)	1 (1.3)	1
POT1 (%)	11 (1.4)	10 (1.5)	1 (1.3)	1
PRDM1 (%)	17 (2.2)	16 (2.3)	1 (1.3)	0.873
PTPN11 (%)	13 (1.7)	11 (1.6)	2 (2.6)	0.849
PTPRB (%)	11 (1.4)	9 (1.3)	2 (2.6)	0.683
PTPRD (%)	19 (2.5)	17 (2.5)	2 (2.6)	1
PTPRK (%)	11 (1.4)	11 (1.6)	0 (0.0)	0.545
PTPRT (%)	14 (1.8)	14 (2.0)	0 (0.0)	0.42

RB1 (%)	11 (1.4)	11 (1.6)	0 (0.0)	0.545
ROBO2 (%)	14 (1.8)	13 (1.9)	1 (1.3)	1
SETD2 (%)	20 (2.6)	18 (2.6)	2 (2.6)	1
SF3B1 (%)	13 (1.7)	12 (1.7)	1 (1.3)	1
TET2 (%)	11 (1.4)	10 (1.5)	1 (1.3)	1
TNC (%)	14 (1.8)	13 (1.9)	1 (1.3)	1
TP53 (%)	**35 (4.6)**	**20 (2.9)**	**15 (19.7)**	**<0.001**
UBR5 (%)	**11 (1.4)**	**7 (1.0)**	**4 (5.3)**	**0.015**
ZFHX3 (%)	16 (2.1)	14 (2.0)	2 (2.6)	1
ZNF521 (%)	11 (1.4)	11 (1.6)	0 (0.0)	0.545

Table S6. Distribution of high-risk cytogenetic abnormalities in the E-MTAB-4032 cohort stratified by risk score group.

Variable	Level	Overall	Standard risk	High risk	p	BH adjusted p-value
n		149	100	49		
del17p (%)	Absent	111 (88.8)	70 (88.6)	41 (89.1)	1	1
	Present	14 (11.2)	9 (11.4)	5 (10.9)		
t(4;14) (%)	Absent	37 (54.4)	20 (45.5)	17 (70.8)	0.074	0.111
	Present	31 (45.6)	24 (54.5)	7 (29.2)		
1q+ (%)	Absent	57 (43.8)	43 (51.2)	14 (30.4)	0.027	0.081
	Present	73 (56.2)	41 (48.8)	32 (69.6)		

BH indicates Benjamini-Hochberg adjusted p-value

Table S7. Distribution of high-risk cytogenetic abnormalities in the GSE136337 cohort stratified by risk score group.

Variable	Level	Overall	Standard risk	High risk	p	BH adjusted p-value
n		265	223	42		
del17 (%)	Absent	249 (94.0)	210 (94.2)	39 (92.9)	0.725	0.90625
	Present	16 (6.0)	13 (5.8)	3 (7.1)		
1q+ (%)	Absent	200 (75.5)	173 (77.6)	27 (64.3)	0.066	0.33
	Present	65 (24.5)	50 (22.4)	15 (35.7)		
t(14;16) (%)	Absent	264 (99.6)	222 (99.6)	42 (100.0)	1	1
	Present	1 (0.4)	1 (0.4)	0 (0.0)		
t(4;14) (%)	Absent	251 (94.7)	212 (95.1)	39 (92.9)	0.471	0.785
	Present	14 (5.3)	11 (4.9)	3 (7.1)		

BH indicates Benjamini-Hochberg adjusted p-value

References

1. Kryukov, F.; Nemec, P.; Radova, L.; Kryukova, E.; Okubote, S.; Minarik, J.; Stefanikova, Z.; Pour, L.; Hajek, R. Centrosome associated genes pattern for risk sub-stratification in multiple myeloma. *J Transl Med* **2016**, *14*, 150, doi:10.1186/s12967-016-0906-9.
2. Danziger, S.A.; McConnell, M.; Gockley, J.; Young, M.H.; Rosenthal, A.; Schmitz, F.; Reiss, D.J.; Farmer, P.; Alapat, D.V.; Singh, A.; et al. Bone marrow microenvironments that contribute to patient outcomes in newly diagnosed multiple myeloma: A cohort study of patients in the Total Therapy clinical trials. *PLoS Med* **2020**, *17*, e1003323, doi:10.1371/journal.pmed.1003323.
3. Broyl, A.; Hose, D.; Lokhorst, H.; de Knegt, Y.; Peeters, J.; Jauch, A.; Bertsch, U.; Buijs, A.; Stevens-Kroef, M.; Beverloo, H.B.; et al. Gene expression profiling for molecular classification of multiple myeloma in newly diagnosed patients. *Blood* **2010**, *116*, 2543-2553, doi:10.1182/blood-2009-12-261032.
4. Barlogie, B.; Tricot, G.; Rasmussen, E.; Anaissie, E.; van Rhee, F.; Zangari, M.; Fassas, A.; Hollmig, K.; Pineda-Roman, M.; Shaughnessy, J.; et al. Total therapy 2 without thalidomide in comparison with total therapy 1: role of intensified induction and posttransplantation consolidation therapies. *Blood* **2006**, *107*, 2633-2638, doi:10.1182/blood-2005-10-4084.
5. Mitchell, J.S.; Li, N.; Weinhold, N.; Forsti, A.; Ali, M.; van Duin, M.; Thorleifsson, G.; Johnson, D.C.; Chen, B.; Halvarsson, B.M.; et al. Genome-wide association study identifies multiple susceptibility loci for multiple myeloma. *Nat Commun* **2016**, *7*, 12050, doi:10.1038/ncomms12050.
6. Zhan, F.; Huang, Y.; Colla, S.; Stewart, J.P.; Hanamura, I.; Gupta, S.; Epstein, J.; Yaccoby, S.; Sawyer, J.; Burington, B.; et al. The molecular classification of multiple myeloma. *Blood* **2006**, *108*, 2020-2028, doi:10.1182/blood-2005-11-013458.
7. van Rhee, F.; Szymonifka, J.; Anaissie, E.; Nair, B.; Waheed, S.; Alsayed, Y.; Petty, N.; Shaughnessy, J.D., Jr.; Hoering, A.; Crowley, J.; et al. Total Therapy 3 for multiple myeloma: prognostic implications of cumulative dosing and premature discontinuation of VTD maintenance components, bortezomib, thalidomide, and dexamethasone, relevant to all phases of therapy. *Blood* **2010**, *116*, 1220-1227, doi:10.1182/blood-2010-01-264333.
8. Skerget, S.; Penaherrera, D.; Chari, A.; Jagannath, S.; Siegel, D.S.; Vij, R.; Orloff, G.; Jakubowiak, A.; Niesvizky, R.; Liles, D.; et al. Comprehensive molecular profiling of multiple myeloma identifies refined copy number and expression subtypes. *Nat Genet* **2024**, *56*, 1878-1889, doi:10.1038/s41588-024-01853-0.
9. Keats, J.J.; Craig, D.W.; Liang, W.; Venkata, Y.; Kurdoglu, A.; Aldrich, J.; Auclair, D.; Allen, K.; Harrison, B.; Jewell, S.; et al. Interim Analysis Of The Mmrf Commpass Trial, a Longitudinal Study In Multiple Myeloma Relating Clinical Outcomes To Genomic and Immunophenotypic Profiles. *Blood* **2013**, *122*, 532-532, doi:10.1182/blood.V122.21.532.532.
10. Heuck, C.J.; Qu, P.; van Rhee, F.; Waheed, S.; Usmani, S.Z.; Epstein, J.; Zhang, Q.; Edmondson, R.; Hoering, A.; Crowley, J.; et al. Five gene probes carry most of the

discriminatory power of the 70-gene risk model in multiple myeloma. *Leukemia* **2014**, *28*, 2410-2413, doi:10.1038/leu.2014.232.

11. Mulligan, G.; Mitsiades, C.; Bryant, B.; Zhan, F.; Chng, W.J.; Roels, S.; Koenig, E.; Fergus, A.; Huang, Y.; Richardson, P.; et al. Gene expression profiling and correlation with outcome in clinical trials of the proteasome inhibitor bortezomib. *Blood* **2007**, *109*, 3177-3188, doi:10.1182/blood-2006-09-044974.
